# Implementation of standard of care CAR-T-cell treatment for patients with aggressive B-cell lymphoma and acute lymphoblastic leukemia in Sweden

**DOI:** 10.1038/s41375-025-02573-y

**Published:** 2025-03-26

**Authors:** Mats Jerkeman, Karin Mellgren, Kristina Sonnevi, Mikael Lisak, Ingemar Lagerlöf, Balazs Kapas, Hanna Sjölund, Jacek Toporski, Hans Hagberg, Stephan Mielke, Gunilla Enblad

**Affiliations:** 1 Skåne University Hospital and Lund University, Lund, Sweden; 2 Sahlgrenska University Hospital, Gothenburg, Sweden; 3 Karolinska University Hospital, Stockholm, Sweden; 4 Linköping University Hospital, Linköping, Sweden; 5 Skåne University Hospital, Lund, Sweden; 6 Karolinska Institutet, Stockholm, Sweden; 7 Uppsala University Hospital, Uppsala, Sweden; 8 Karolinska Institutet and University Hospital, Stockholm, Sweden

**Keywords:** Immunotherapy, Lymphoma

## Introduction

Treatment with CAR-T cells (chimeric antigen receptor T-cells) has provided a paradigm shift in the treatment of patients with relapsed and refractory aggressive B-cell lymphoma (r/r ABCL) and acute lymphoblastic leukemia (r/r ALL). Sweden, through Uppsala University Hospital, was first in Europe to initiate an academic CAR-T trial for patients with B-cell malignancies [1, 2].

In 2017, the first two commercially available CAR-T-cell products for the treatment of ABCL were approved [3, 4]. The registration studies showed that CAR-T cell treatment can lead to long-term remission in up to about 40% of patients with r/r ABCL. In the same year tisagenlecleucel (tisa-cel) was approved for patients with r/r B-ALL in children and young adults, showing long term remission in around 50% of patients [5]. Tisa-cel was approved in Sweden for R/R pre-B-ALL in children or adults ≤25 years of age in 2019. Axicabtagene ciloleucel (axi-cel) was approved in the same year for ABCL, and six hospitals are so far accredited to use this product. All potential cases are discussed at multidisciplinary national conferences, applying the EHA/EBMT recommendations [6].

More recently, real life data on the outcome of CAR T cell patients have been gathered from several countries [7–10]. Awaiting the results of the post-authorization studies, we would like to publish and highlight the promising Swedish real-life experience of CAR-T cell therapy with axi-cel and tisa-cel in the two initial available indications.

## Methods

All patients undergoing leukapheresis or their guardians provided written informed consent to collect, report and analyze their data (Dnr 2023-00179-02). One guardian withdrew the consent. This data has been excluded from this analysis. Anonymized data on demographics, diagnosis, treatment lines, time from treatment to recurrence, maximum degree of cytokine release syndrome (CRS) and immune effector-cell associated neurotoxicity syndrome (ICANS) graded according to the American Society for Transplantation and Cellular Therapy guidelines were collected, as well as response to axi-cel at 30 days by PET-CT. Date of progression, last follow-up date, reinfusion and date of death were recorded. Progression-free survival (PFS) and overall survival were calculated according to Kaplan-Meier.

## Results

### Axi-cel in ABCL

#### Patients

During the period December 2019 to January 2024, 101 patients with ABCL underwent leukapheresis for production of axi-cel, due to relapse or progression after 2 or more previous treatment lines at 5 centers in Sweden. A total of 93 patients have undergone treatment. Eight patients were excluded from treatment due to rapid disease progression (*n* = 7), or lack of compliance (*n* = 1). For patient characteristics, see Table 1. The median follow-up time from CAR-T cell infusion is 15 months.

**Table 1:** Patient characteristics – aggressive B-cell lymphoma.

Age (median, min, max)	63 years (17, 78)	
Sex	Men	60 (65%)
	Women	33 (35%)
Diagnosis	DLBCL	61 (66%)
	High grade B-cell lymphoma	9 (10%)
	Transformed follicular lymphoma	13 (14%)
	Primary mediastinal large B-cell lymphoma	4 (4%)
	Other aggressive B-cell lymphomas	6 (7%)
Treatment line	2	12 (13%)
	3	45 (48%)
	4	24 (26%)
	>4	12 (13%)
Recurrence patterns	Primary refractory disease	34 (37%)
	Recurrence <12 months after stopping treatment	25 (27%)
	Recurrence ≥12 months after discontinuation of treatment	34 (37%)
	CNS involvement at relapse	10 (11%)
Year of CAR-T infusion	2019	1
	2020	8
	2021	23
	2022	31
	2023	29
	2024	1
Cytokine release syndrome (CRS) Maximum grade	0	5 (5%)
	1	42 (46%)
	2	44 (48%)
	3	1 (1%)
	4	0
Immune effector-cell associated neurotoxicity syndrome (ICANS) Maximum grade	0	52 (56%)
	1	11 (12%)
	2	15 (16%)
	3	8 (9%)
	4	6 (7%)

#### Bridging

Most patients, 88 (95%), received bridging therapy. Radiotherapy was used in 30 patients. Among systemic therapies, polatuzumab vedotin was the most commonly used agent (*n* = 38), followed by combinations including gemcitabine (*n* = 23) and platinum (*n* = 22). Bendamustine combinations were used in 12 patients, all in combination with polatuzumab vedotin and a CD20 antibody. Four patients received both radiotherapy and systemic therapy.

#### Toxicity

Only one patient (1%) experienced grade ≥3 CRS, and 16% grade 3-4 ICANS during the first 30 days post infusion. (Table 1). Data on hematological toxicity was not systematically collected.

#### Efficacy

87 out of 93 patients were evaluable for response at 30 days. Complete remission was achieved in 57 (66%) patients, 11 (13%) partial remission, 6 (7%) stable disease, and 13 (15%) progressive disease.

**Fig. 1:**
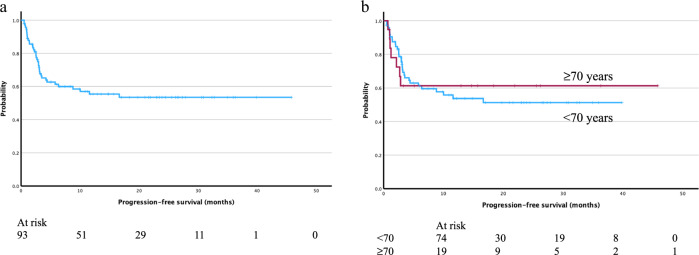
Outcomes for patients treated with axi-cel in Sweden. Progression-free survival (**a**) for all patients treated with axi-cel in Sweden, **b** divided into patients <70 years and ≥70 years at time of treatment.

For infused patients, the estimated PFS after 12 months was 53% (Fig. 1a). In patients above 70 years of age, a tendency towards better PFS was observed compared to younger patients (Fig. 1b). Patients with secondary CNS involvement showed inferior outcome (Fig. 1Sa). We also observed an association between higher grade of ICANS and improved PFS (Fig. 1Sb). There was no difference in PFS between patients with late relapse vs refractory to previous lines of treatment.

Twenty-six patients have died, most due to lymphoma (*n* = 21, 81%). Five patients died due to treatment related toxicity (intraabdominal bleeding, bowel perforation, sepsis, aspergillosis, heart failure, 1 each). No patient has died due to CRS or ICANS. Estimated overall survival at 24 months is 67% (Fig. 1Sc).

## Tisa-cel in precursor-B-ALL

During the period December 2019 to June 2023, a total of 13 pediatric and young adult patients underwent leukapheresis for production of tisa-cel, due to relapse after stem cell transplantation or refractory disease. One guardian to a patient withdrew the consent, so that 12 patients remain available for analysis. For patient characteristics see Table 1S. The median follow-up time is 26.8 months after CAR-T infusion.

### Bridging

Most patients, 11 (92%), received bridging therapy with different combinations of chemotherapy before CAR-T infusion.

#### Toxicity

One patient (8%) experienced grade 3 CRS, and three patients (25%) grade 3-4 ICANS during the first 30 days post infusion. One of the patients with ICANS suffered from a subsequent intracranial bleeding. Data on hematological toxicity was not systematically collected.

#### Efficacy

12 out of 12 patients were evaluable for response at 30 days. Complete remission was achieved in 11 (92%) patients. Six patients (50%) suffered from relapse of the initial leukemia after the first CAR-T infusion with at a median time to relapse of 3.7 months. Four patients received a second infusion of the remaining product, three of them due to relapse of the leukemia and one due to increasing B-cells, suspected to indicate loss of CAR-T cells. All four of the twice infused patients suffered a new relapse after the second infusion of CAR-T cells, and all of these were rescued with a later stem cell transplantation. In total, five patients underwent a stem cell transplantation after CAR-T treatment, three due to relapse after second CAR-T infusion and two for other reasons. Nine patients suffered from a CRS after CAR-T infusion; five grade 1, four grade 2 and one grade 3. Three patients had ICANS post-CAR-T; one patient grade 3 and two patients grade 4. One of the patients with ICANS suffered from a subsequent intracranial bleeding.

In total four patients died after CAR-T treatment, three from relapse of the leukemia and one for treatment related reasons.

## Discussion

In this report, we have summarized our overall real-life experience of CAR-T-cell treatment of patients with r/r ABCL and B-ALL. Recommendations on treatment are made in all cases at national multidisciplinary conferences, with participation from all accredited clinics. In most regions, a positive recommendation from the national conference is also a prerequisite for approval of payment.

The basis for the approval of axi-cel at the European Medicines Agency (EMA) is the ZUMA-1 trial, where 111 patients with ABCL who had received at least two previous regimens were included [3]. The frequency of grade 3–4 CRS was 11% in the ZUMA-1. In our dataset, only 1% CRS grade 3 was encountered. Moreover, in ZUMA-1, 32% of treated patients experienced grade 3–4% ICANS, compared to 16% in our material. The reasons for the lower frequency may be stricter selection of patients, more intensive bridging therapy or that the experience of treating these conditions has improved over time. In terms of treatment efficacy, complete remission was seen in 58% in ZUMA-1, and PFS after 12 months was 44%.

The efficacy and safety of axi-cel has also been studied “real world”. In a report by the US Lymphoma CART Consortium, including 275 treated patients from 17 US centers, PFS after 12 months was 47%, and grade ≥3 CRS and ICANS, 7% and 31%, respectively [11]. Data from the German lymphoma registry, for 173 patients treated with axi-cel, show a level of grade 3-4 CRS and ICANS that are more comparable to the Swedish – 10 and 16%, respectively. PFS after 12 months in this material was 35% [7].

In summary, the Swedish data appear favorable in an international comparison. The encouraging results are most likely a result of national multidisciplinary conferences and cooperation between centers in addition to a strict selection of patients.

We report an association between higher grade ICANS and improved outcome, a relationship that may deserve further study. Possibly, the peak CAR-T cell level is related both to response and risk of ICANS [12]. Outcome for elderly patients (>70 years) was favorable, possibly due to more strict selection of patients within this age group.

CAR-T cell therapy has recently been introduced in patients with ABCL as second-line treatment in patients with early recurrence (within 12 months), based on two randomized phase 3 studies, comparing CAR-T with today’s standard of care, high-dose chemotherapy with autologous stem cell support, showing prolonged survival with CAR-T [13, 14]. It remains to be seen if outcome in this population will be superior to that of patients treated at a later stage.

Our experience with tisa-cel in pre-B ALL is still limited. With 6 of 13 (46%) patients free from relapse after 2 years, our data are in line with real world studies in US [15].

CAR-T has thus been established as the standard treatment for lymphoma and pre-B ALL in Sweden. It is a potentially curative treatment, and it is of the utmost importance that all patients in the country continue to have equal access to this therapy.

## Supplementary information

Supplementary information
