# Supplementary material for: Implementation of standard of care CAR-T-cell treatment for patients with aggressive B-cell lymphoma and acute lymphoblastic leukemia in Sweden
Source: Leukemia. 2025 Mar 26;39(5):1256–9. doi: 10.1038/s41375-025-02573-y (PMC12055569; doi:10.1038/s41375-025-02573-y)
Supplement: Supplementary file 1 — Supplementary information [file 41375_2025_2573_MOESM1_ESM.pdf]

**Supplementary information**

**Table 1S**

**Patient characteristics, acute lymphoblastic leukemia**

| | | |
|---|---|---|
| Age (median, min, max) | 15.6 years (2.1, 23.6) | |
| Sex | Men | 10 (83%) |
| | Women | 2 (17%) |
| Diagnosis | BCP-ALL (>1 y, non-DS) | 12 (100%) |
| | CNS involvement before CAR-T infusion | 2 (17%) |
| Indication for CAR-T | refractory disease | 4 (33%) |
| | 2nd or higher relapse | 1 (8%) |
| | relapse post SCT | 7 (58%) |
| Relapse after CAR-T infusion | No of patients | 6 (50%) |
| | Median time to relapse after CAR-T infusion (min, max) | 3.7 months (2.5, 9.2) |
| Site of relapse | Extramedullary disease including CNS | 3 (25%) |
| | Bone marrow | 3 (25%) |
| Post CAR-T treatment | $2^{nd}$ CAR-T infusion | 3 (25%) |
| | HSCT post CAR-T | 5 (42%) |

HSCT=Hematopoietic cell transplantation

**Figure 1S**

Outcomes for patients treated with axi-cel in Sweden. Progression-free survival (a) divided into patients with and without CNS involvement at time of treatment, (b) in relation to maximum grade immune effector cell associated neurotoxicity (ICANS) (c) overall survival in all patients treated.

a.

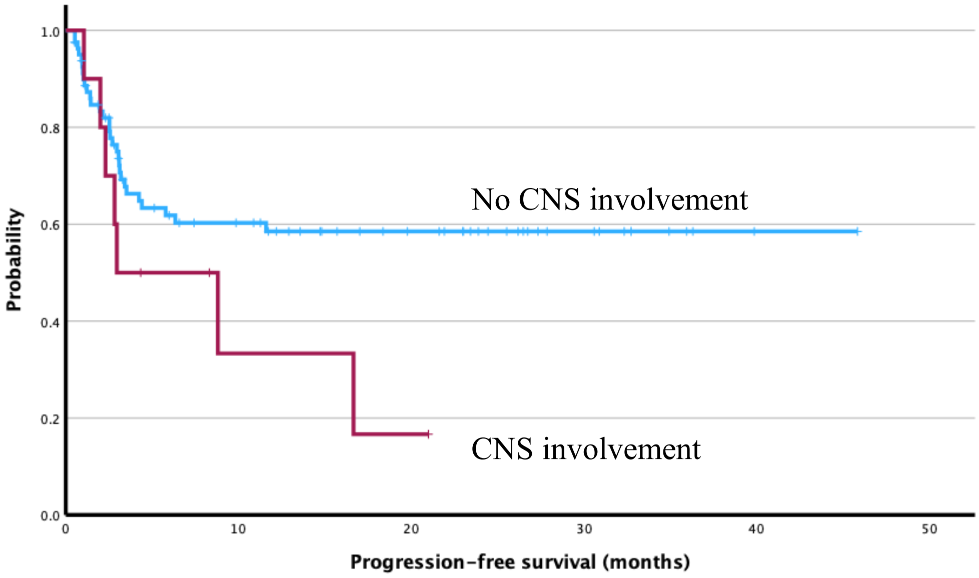


| | At risk | | | | |
|---|---|---|---|---|---|
| No CNS | 81 | 36 | 23 | 10 | 1 |
| CNS | 10 | 2 | 1 | 0 | 0 |

b.

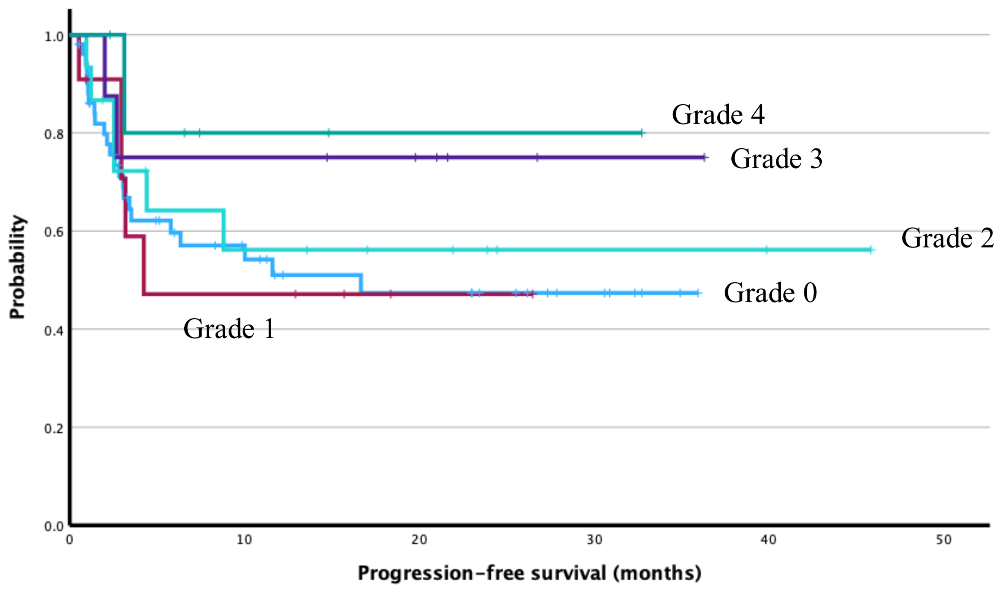


| | At risk | | | | |
|---|---|---|---|---|---|
| Grade 0 | 52 | 20 | 13 | 6 | 0 |
| Grade 1 | 11 | 4 | 1 | 0 | 0 |
| Grade 2 | 15 | 7 | 5 | 2 | 1 |
| Grade 3 | 8 | 6 | 4 | 1 | 0 |
| Grade 4 | 6 | 2 | 1 | 1 | 0 |

c

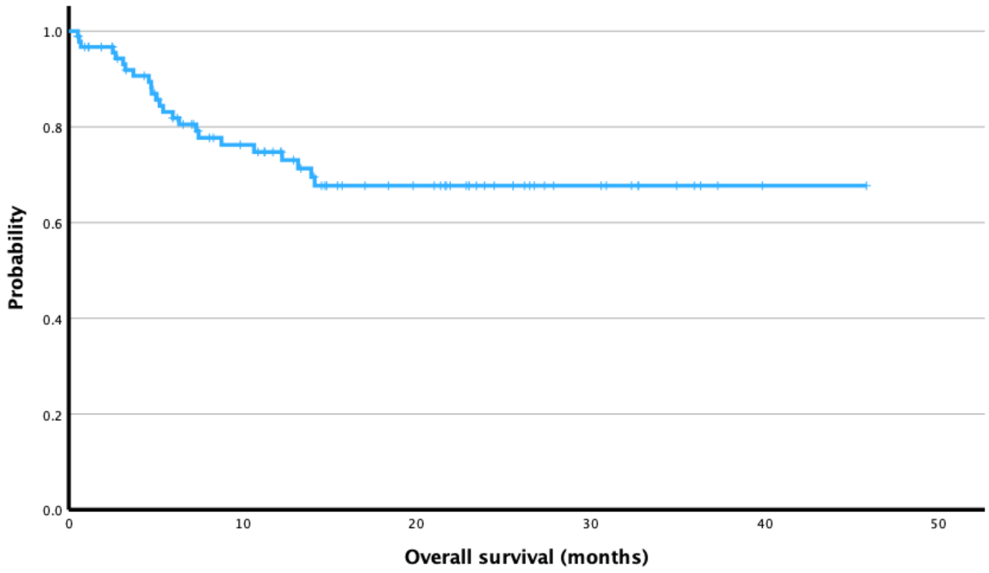


| At risk | | | | | |
|---|---|---|---|---|---|
| 93 | 51 | 29 | 11 | 1 | 0 |
